# Fitness consequences of redundant cues of competition in male *Drosophila melanogaster*

**DOI:** 10.1002/ece3.6293

**Published:** 2020-05-04

**Authors:** Alice A. Dore, Amanda Bretman, Tracey Chapman

**Affiliations:** ^1^ School of Biological Sciences University of East Anglia Norwich UK; ^2^ School of Biology Faculty of Biological Sciences University of Leeds Leeds UK

**Keywords:** behavioral plasticity, fruit fly, sperm competition

## Abstract

Phenotypic plasticity can allow animals to adapt their behavior, such as their mating effort, to their social and sexual environment. However, this relies on the individual receiving accurate and reliable cues of the environmental conditions. This can be achieved via the receipt of multimodal cues, which may provide redundancy and robustness. Male *Drosophila melanogaster* detect presence of rivals via combinations of any two or more redundant cue components (sound, smell, and touch) and respond by extending their subsequent mating duration, which is associated with higher reproductive success. Although alternative combinations of cues of rival presence have previously been found to elicit equivalent increases in mating duration and offspring production, their redundancy in securing success under sperm competition has not previously been tested. Here, we explicitly test this by exposing male *D. melanogaster* to alternative combinations of rival cues, and examine reproductive success in both the presence and absence of sperm competition. The results supported previous findings of redundancy of cues in terms of behavioral responses. However, there was no evidence of reproductive benefits accrued by extending mating duration in response to rivals. The lack of identifiable fitness benefits of longer mating under these conditions, both in the presence and absence of sperm competition, contrasted with some previous results, but could be explained by (a) damage sustained from aggressive interactions with rivals leading to reduced ability to increase ejaculate investment, (b) presence of features of the social environment, such as male and female mating status, that obscured the fitness benefits of longer mating, and (c) decoupling of behavioral investment with fitness benefits.

## Graphical abstract

Phenotypic plasticity can allow animals to adapt their behavior, such as their mating effort, to their social and sexual environment. However, this relies on the individual receiving accurate and reliable cues of the environmental conditions. Here, we explicitly test this by exposing male *Drosophila melanogaster* to alternative combinations of rival cues and examining reproductive success in both the presence and absence of sperm competition. The results supported previous findings but there was no evidence of reproductive benefits accrued by extending mating duration in response to rivals.

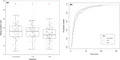

## 1 INTRODUCTION

Many animals exhibit plasticity in their reproductive behavior and/or reproductive investment in response to the other organisms around them, allowing them to allocate resources across mating opportunities in order to maximize lifetime reproductive success (Dewsbury, 1982; Gage, 1995; Kokko & Rankin, 2006; Parker, 1982; Rodriguez, Rebar, & Fowler‐Finn, 2013; Wedell, Gage, & Parker, 2002). However, in order for plasticity to be adaptive, cues that confer accurate, reliable, and robust information on the current conditions must be received (Auld, Agrawal, & Relyea, 2010; DeWitt, Sih, & Wilson, 1998). One way in which the information conferred by environmental cues may be made more robust is through the receipt of multicomponent or multimodal (complex) cues. Cues can be categorized as multicomponent if they are received via one sensory modality or as multimodal if the components are received through multiple sensory modalities (Candolin, 2003; Hebets & Papaj, 2005). Here, we use “complex cue” as a general term for multicomponent and multimodal cues, and “cue component” as an umbrella term for the separate modalities or components that comprise the complex cue. Redundancy among cue components can mean that even if one component is lost or compromised, the overall information within any message can remain intact (Bro‐Jorgensen, 2010; Johnstone, 1996). This suggests that receiving alternative combinations of cue components should elicit equivalent phenotypic changes and equivalent associated fitness benefits. However, redundancy may also be incomplete, whereby separate components relay partially overlapping, but not identical, information about the environment (Bretman, Westmancoat, Gage, & Chapman, 2011b; Dore et al., 2018). In this scenario, altering the combination of cue components to which an individual is exposed may result in subtle effects on subsequent phenotypes, with associated fitness consequences.

Both multimodal and multicomponent cues are abundant in mating systems and may often be subject to sexual selection via their effects on both the signaller and the receiver (Bro‐Jorgensen, 2010). The separate elements of a cue may be entirely or partially redundant, convey distinct information, or interact—for example, one cue component may grab the attention of the receiver while the others convey information (Bro‐Jorgensen, 2010). In field crickets (*Teleogryllus oceanicus*), female responses to male acoustic performances are mediated by the presence of CHCs, suggesting that mate choice is dependent on a multimodal signal encompassing auditory and olfactory components (Bailey, 2011). In this instance, the two modalities are found to interact and may increase the amount of information that can be perceived. Male wolf spiders (*Schizocosa ocreata*) also perform multimodal courtship displays which consist of visual and seismic components, but in this case, the cue components appear to be redundant. On substrates that weakened the effectiveness of seismic components of courtship males were found to increase their use of visual signals, suggesting that the two cue components can act as “backups” to ensure the signal can be received across varying environments (Gordon & Uetz, 2011). An example of complex mating signals being contained within one sensory modality can be found in swordtail fish (*Xiphophorus nigrensis*), in which females respond to multicomponent male visual displays. Male size and courtship vigor did not have an additive effect on female preference, but females responded more quickly to males when both components were increased (Reding & Cummings, 2017). This offers further evidence that cue components can interact to influence the mating decisions of the receiver. Overall, these examples demonstrate that identifying complex cues and understanding how the components overlap or interact can shed light on complex mating behaviors and how these behaviors can vary across environments.

A well‐studied example of redundant, multimodal signaling comes from the reproductive behavior of male *Drosophila melanogaster*, which offers excellent potential for studying how redundancy in cue components can affect plastic behavior. Male *D. melanogaster* express behavioral plasticity, whereby individuals exposed to rival males will subsequently mate for longer and increase their transfer of some seminal fluid proteins, in comparison with males housed alone (Bretman, Fricke, & Chapman, 2009; Wigby, Sirlot, et al., 2009). Extended matings following exposure to rivals are reported to be associated with increased paternity share (Bretman et al., 2009). However, exposure to rivals over a male's whole lifetime results in the expression of reproductive costs later in life (Bretman et al., 2009; Bretman, Westmancoat, Gage, & Chapman, 2013b). The behavioral response of male *D. melanogaster* to rival males is highly sensitive to the level of competition and can rapidly be reversed upon the removal of competition (Bretman, Westmancoat, Gage, & Chapman, 2012). Male *D. melanogaster* can detect rival males via three sensory cues: tactile, olfactory, and auditory (Bretman, Westmancoat, et al., 2011). Males exposed to any two of these cues in combination, or all three, responded with equivalent extensions to subsequent mating duration. The finding that removing any one cue of rival presence does not prevent the male from responding suggests that there is redundancy in how these cues are processed. This redundancy may confer robustness in responses to the social environment, which can be complex and rapidly variable (Bretman, Gage, & Chapman, 2011a; Dore et al., 2018; Greenspan, 2012; Kasumovic, Bruce, Andrade, & Herberstein, 2008). Although male *D. melanogaster* with one sensory cue removed were able to respond to rivals, a longer period of exposure was required to elicit the longer mating response, compared to males with all cues intact (Rouse & Bretman, 2016). Furthermore, the combination of cues a male is exposed to has a role in species recognition of rivals (Bretman, Rouse, Westmancoat, & Chapman, 2017). This suggests that there may be incomplete redundancy in how the cues of rival presence are processed in order to produce the behavioral response.

In addition to eliciting equivalent behavioral responses, perceiving any two of the three rival cues appears to result in comparable increases in the number of offspring fathered (Bretman, Westmancoat, et al., 2011). However, thus far this has only been tested in the absence of realized sperm competition; hence, an important facet of the fitness consequences of responding to rival males is not yet known. This is the omission we tackle in this study. Determining whether males that have any one sensory cue systematically removed achieve equivalent success in sperm competition is important as it is expected to increase our understanding of the fitness benefits and potential costs of redundancy in general.

We explicitly tested the fitness equivalence of receiving alternative cues of rival presence under sperm competition, to investigate further whether these cues show complete redundancy. Male *D. melanogaster* were exposed to intact rivals or those subjected to a physical manipulation that removed the auditory cue of rival presence. We focused on testing auditory cue removal as this could be fully controlled (removal of tactile and olfactory cues produced off‐target effects on male behavior; Appendix 1, 2; Figures S1, S2). Our rationale for focusing our experiments on just this single cue removal as the exemplar was that previous tests reported that all cues were equivalent with respect to the subsequent behavioral and fitness outcomes (Bretman, Westmancoat, et al., 2011). Thus, the effects of removing the auditory cue can inform our understanding of the redundancy of all three key cues.

Males exposed to the full repertoire of cues (auditory+tactile+olfactory) and those with one cue removed (tactile+olfactory) were both predicted to show equivalent extension in mating duration and increase in noncompetitive paternity compared to males that had no rival exposure, as identified by Bretman, Westmancoat, et al. (2011). In addition, we predicted that males exposed to either of the above combinations of rival cues would achieve an equivalent increase in competitive paternity when the female subsequently remated, relative to males kept without rivals. This would support the idea that the cues of rival presence perceived by male *D. melanogaster* are redundant.

## 2 MATERIALS AND METHODS

### 2.1 General methods

Experiments were conducted in a 25°C humidified room held under a 12 hr light: 12 hr dark cycle. Flies were maintained in 75 × 25 mm glass vials containing 7 ml sugar–yeast–agar (SYA) medium (100 g brewer's yeast, 50 g sucrose, 15 g agar, 30 ml Nipagin (10% solution), 3 ml propionic acid, and 0.97 L water per liter of medium). Wildtype flies were sampled from the Dahomey population (Bretman et al., 2009). Females were allowed to oviposit on agar–grape juice plates (50 g agar, 600 ml red grape juice, 42 ml Nipagin (10% solution), and 1.1 L water). All larvae were reared under a controlled density of 100 per vial to minimize variation in adult body size (Miller & Thomas, 1958). Adults were collected and separated by sex within 8 hr of eclosion to ensure virginity and stored 10 per vial. All male and female flies were age‐matched, between sexes and across treatments.

### 2.2 Sensory cues removal

Each male was randomly assigned into one of three treatments: housed with a rival male with all sensory cues intact (+ all), housed with a rival with the auditory cue removed (+ no sound), or housed alone (− all). The experiment was repeated in two independent replicates, which were pooled for analysis. The auditory cue of rival presence was removed by using a physical manipulation in which the wings of the rival males were removed under CO_2_ anesthesia, preventing them from producing the song that signals their presence to competitors. To control for handling, the rival males in the +all treatment were also subjected to CO_2_ anesthesia and the tips of their wings were clipped, allowing identification of the focal male but not affecting the capacity of rival males to produce song (Ehrman, 1966). The focal and rival males in the + no sound and + all treatments were housed together in a single SYA vial. The males in the − all treatment were housed alone in a vial. Focal males were maintained in their respective treatments for 3 days.

### 2.3 Effect of cue removal on responses to rivals and reproductive success and sperm competitive ability

Virgin wildtype females were transferred to individual vials of SYA 1 day prior to mating. Each treatment male was introduced to a female directly from their rival treatments by using aspiration. Latency to mate (the time from when the male was introduced to when mating began) and mating duration were recorded to the nearest minute. Pairs that did not mate within 3 hr were discarded. Males were removed from the vials by aspiration shortly after mating finished to prevent any rematings. Females were allowed to oviposit in a first set of vials for 24 hr, following which each female was transferred to a fresh vial. The first set of vials was then incubated, and offspring that emerged from them were counted.

Approximately 24 hr after the first mating, females were given the opportunity to mate a second time, to males with a “stubble” (*Sb*) mutation. *Sb* mutant individuals are identifiable by the shorter, thicker bristles on the back of the thorax (Overton, 1967), allowing for offspring paternity to be determined by eye. *Sb* males came from a *Sb^1^* stock which had been backcrossed into the Dahomey wild type background at least four times. The proportion of females that remated was recorded, and as in the first‐mating assay, the latency and duration of the rematings were recorded to the nearest minute. Pairs that did not mate within 3 hr were discarded. Males were removed shortly after mating. Females were allowed to oviposit in the vials for 24 hr, after which they were discarded. The vials were retained and incubated. Offspring that developed from eggs laid following the second mating had mixed paternity, some being fathered by the first (treatment) male and some by the second *Sb* male. Paternity was thus determined by the presence of the *Sb* phenotype allowing us to calculate the proportion of the offspring fathered by the first (treatment) male (P1) and by the second (*Sb* competitor) male (P2).

### 2.4 Statistical analysis

Statistical analyses were carried out in R v 3.4.2 (R Core Team, 2016). The data from the two replicates were pooled, then analyzed and plotted as one dataset with replicate as a fixed factor. Mating latency data from the first and second mating were analyzed using cox proportional hazards models. Shapiro–Wilk and Levene's tests were used to assess whether mating duration and offspring count data were normally distributed and whether variances were equal across treatments, respectively. Where the data were normally distributed or could be transformed to fit a normal distribution they were analyzed using linear models.

Offspring counts from the first mating in both blocks were zero‐inflated, so were analyzed using hurdle models. The number of zero offspring counts in each treatment and the nonzero counts was manually separated. The number of zeroes was analyzed with a binomial generalized linear mixed model. Where the nonzero offspring counts were normally distributed or could be transformed to fit a normal distribution, they were analyzed with a linear mixed model. Otherwise, nonzero counts were analyzed using a generalized linear mixed model with a Poisson distribution and a log link. In order to infer the effect of treatment on overall offspring counts, including zeroes and nonzeroes, Kruskal–Wallis tests were used. The proportion of offspring produced following the second mating that was fathered by the treatment male (P1) was analyzed as a dual response variable using a binomial generalized linear mixed model with a logit link.

As a significant effect of treatment was found on first‐mating duration, pairwise differences between groups were determined using post hoc Tukey's tests with the “multcomp” package (Hothorn, Bretz, & Westfall, 2008). Pairwise comparisons between treatment groups of the number of offspring (nonzero counts) produced after the first mating were made using Wilcoxon's test. The proportion of paternity achieved by the first male after the second mating was compared between treatments using two‐sample proportion *z* tests. Across all analyses, *p*‐values were adjusted using the Benjamini–Hochberg procedure.

## 3 RESULTS

**Figure 1:**
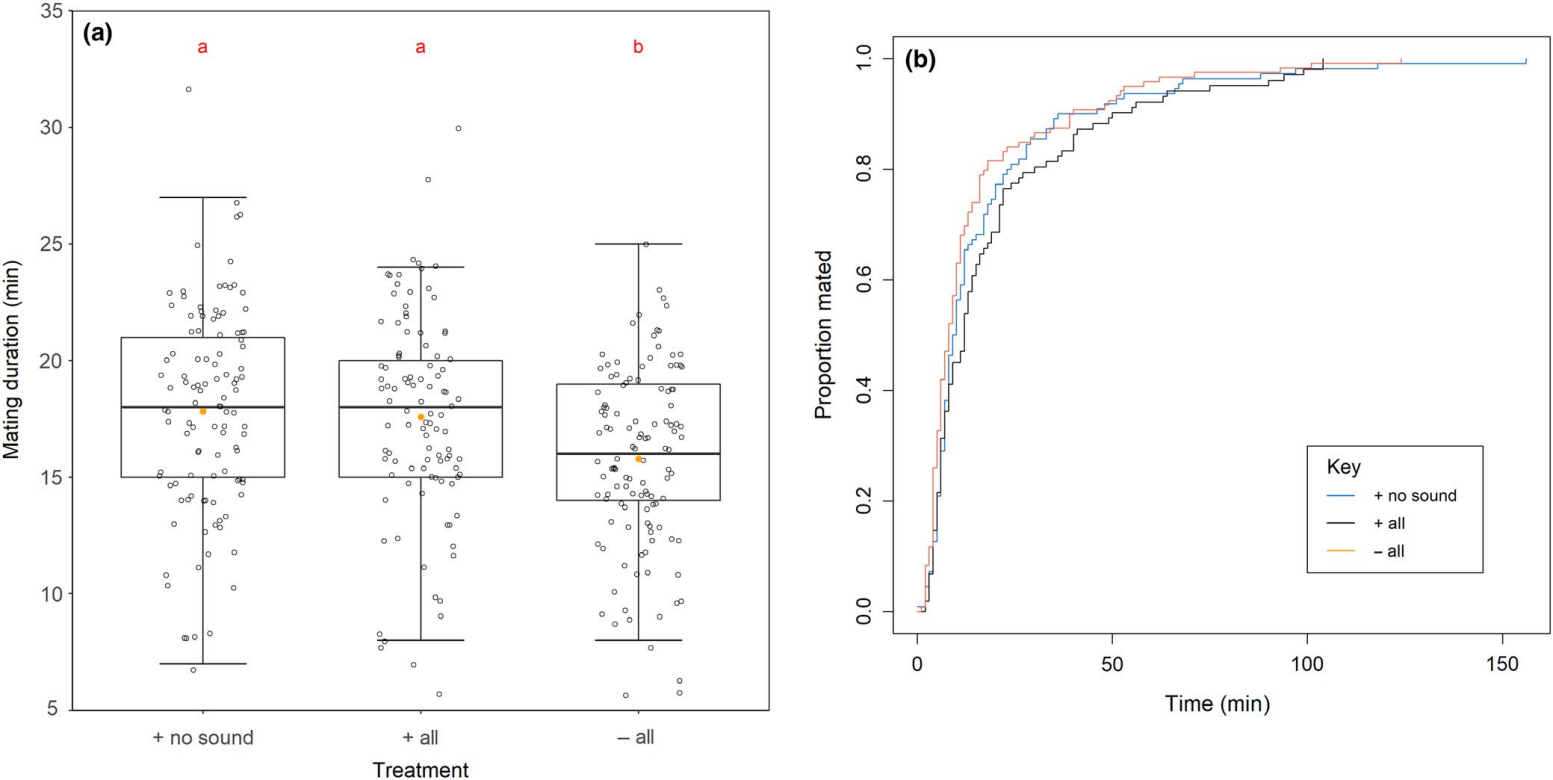
The (a) mating duration and (b) mating latency of males either exposed to a rival male with the auditory cue removed (+ no sound), all cues intact (+ all), or housed alone without rival exposure (− all). (a) Boxplot shows interquartile range and median with raw data points also plotted. Orange dots indicate means; red letters indicate significance of pairwise differences. (b) The proportion of males in each treatment (blue = + no sound; black = + all; orange = − all) that had mated over time

The mating duration of the treatment males was significantly affected by the cues of rival presence to which males were exposed (*F* = 7.62, *df* = 2 & 329, *p* = .00049; Figure 1a). Males exposed to the full repertoire of rival cues (+ all; *p* = .019) and those that had been exposed to rivals with the auditory cue removed (+ no sound; *p* = .0049) both significantly extended mating duration relative to males that had not encountered rivals. This is consistent with previous research showing that removing one cue signaling the presence of competitors does not impede a male's ability to respond by significantly increasing mating duration.

The effect of the cues of rival presence the male was exposed to did not significantly affect mating latency (χ^2^ = 5.56, *df* = 2, *p* = .16; Figure 1b). The influence of the experimental block in which the male was tested was borderline nonsignificant (χ^2^ = 5.63, *df* = 1, *p* = .051), which taken with previous findings suggests that mating latency in response to rivals does not show high repeatability (Bretman et al., 2009; Bretman, Westmancoat, & Chapman, 2013a; Bretman, Westmancoat, Gage, et al., 2013).

**Figure 2:**
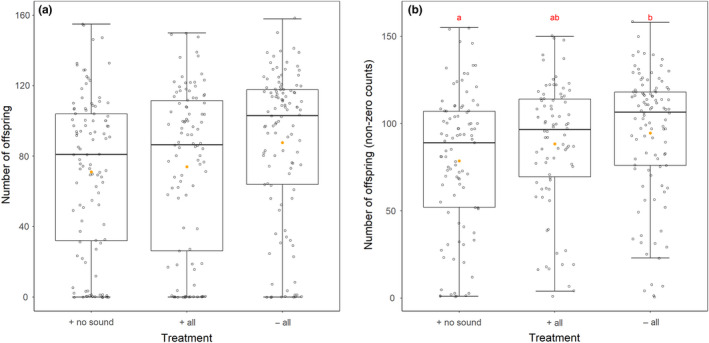
The number of offspring fathered in 24 hr following a single mating, (a) with all data included and (b) with zero counts removed. Focal males were either exposed to a rival male with the auditory cue removed (+ no sound), all cues intact (+ all), or housed alone without rival exposure (− all). Boxplots as in Figure 1

Following mating with the treatment males, females were allowed to oviposit for 24 hr before remating. This allowed us to quantify the reproductive success of the treatment males in the absence of sperm competition before remating. A Kruskal–Wallis test showed that the number of offspring produced was significantly affected by rival cues to which males were exposed (χ^2^ = 11.00, *df* = 2, *p* = .018; Figure 2a). As the offspring count data were zero‐inflated, a hurdle model was then used in which zeroes and nonzeroes were separated and modeled. The number of zeroes in offspring counts was not significantly predicted by the rival cues the male was exposed to (χ^2^ = 4.50, *df* = 2, *p* = .23). Nonzero offspring counts were significantly influenced by an interaction between male treatment and replicate (χ^2^ = 8.52, *df* = 2, *p* = .046; Figure 2b) suggesting that while treatment significantly affected the number of offspring produced, this effect varied across replicates and may not show high repeatability. Contrary to predictions, pairwise testing showed that males who were not exposed to any rival cues (− all) fathered significantly more offspring than those exposed to rivals with the auditory cue removed (+ no sound; *w* = 6,168, *p* = .0049). Males exposed to the full repertoire of rival cues (+ all) fathered an intermediate number of offspring.

Females were given the opportunity to remate to an *Sb* male 24 hr after their first matings, in order to assess the reproductive success of the first‐mating treatment males under sperm competition. The proportion of females that remated was low across treatments (+ no sound: 38%; + all: 28%; − all: 35%) and was not significantly affected by the rival cues the focal males were exposed to (χ^2^ = 2.38, *df* = 2, *p* = .48). Neither latency to remate (χ^2^ = 2.60, *df* = 2, *p* = .27; Figure S3) nor remating duration (*F* = 1.12, *df* = 2 & 110, *p* = .48; Figure S4) were predicted by the rival cues to which the first males were exposed.

**Figure 3:**
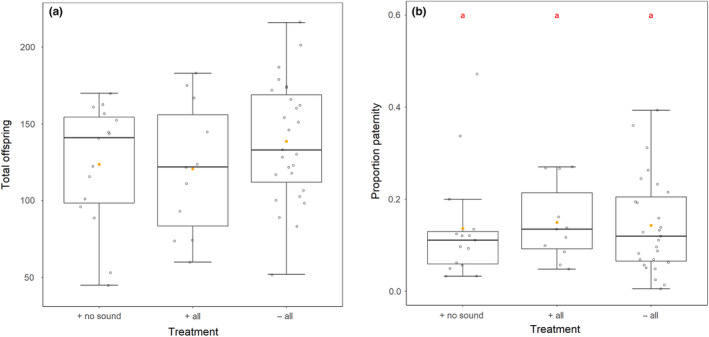
(a) The total number of offspring that were produced following a second mating with a *Sb* mutant male, and (b) the proportion of these offspring that were fathered by the first, focal male (P1). Focal males were either exposed to a rival male with the auditory cue removed (+no sound), all cues intact (+all), or housed alone without rival exposure (−all). Boxplots as in Figure 1

The proportion of offspring produced in the 24 hr following the second mating that was fathered by the first (focal) male was significantly affected by an interaction between the rival cues the male was exposed to and experimental replicate (χ^2^ = 63.24, *df* = 2, *p* < .001; Figure 3). This suggests an effect of the rival cues detected by the first male on his success in sperm competition, but that this effect may vary significantly across replicates. Pairwise comparisons did not reveal any significant differences between treatment groups. This is contrary to the expectation that males exposed to rivals, either with all cues intact or with the auditory cue removed, would show equivalent increases in sperm competitiveness, compared to males that had not encountered rivals. Finally, the total number of offspring produced by the female following the second mating was not significantly affected by the treatment of the first male (*F* = 0.299, *df* = 2 & 55, *p* = .80).

## 4 DISCUSSION

Overall the results supported the previous finding of redundancy of cues of *D. melanogaster* male rival presence. However, this redundancy may be incomplete due to differences in the ways these cue components are perceived and processed. Unexpectedly, no fitness benefits of extending mating duration in response to rivals were observed. This suggests that there may not be a simple, direct relationship between behavioral investment in mating and fitness consequences.

### 4.1 Redundancy of cues of *D. melanogaster* rival presence

The results supported the previous finding that removing one cue of rival presence does not affect the ability of male *D. melanogaster* to detect rivals and respond to them by extending their subsequent mating duration (Bretman, Westmancoat, et al., 2011). Males exposed to a rival with the auditory cue removed showed equivalent mating duration to males housed with rivals with all cues intact, and both groups of males mated for significantly longer than males that had not encountered a competitor. This supports the conclusion that alternative cue combinations elicit equivalent behavioral responses. Although the auditory, olfactory, and tactile cues of rival presence appear to be interchangeable in terms of the behavioral response they elicit (Bretman, Westmancoat, et al., 2011), subsequent research suggests that the processing of these cues is not fully redundant (Rouse & Bretman, 2016) and may be underpinned by alternative pathways of gene expression (Dore et al. unpublished data). Our results support the idea that there is at least partial redundancy in how cues indicating the presence of rivals are processed by the receiving male (Bretman, Westmancoat, et al., 2011).

The way in which multiple cues are perceived and processed is likely to be related to social learning, whether these cues are redundant or confer information about different components of the social environment. Learning relies on cues being perceived, stored, and compared to new environmental information (Bailey & Zuk, 2009; Dukas, 2008). Understanding which cues are important for influencing social behavior, and how they lead to a behavioral outcome, may in turn increase understanding of the processes underlying social learning. A form of long‐term memory has been found to be necessary for male *D. melanogaster* males to respond to rivals by adjusting mating duration (Rouse, Watkinson, & Bretman, 2018). It has been suggested that the timing of this response is important, on the basis that a minimum period of exposure to rivals of 24 hr is required to elicit a response (Bretman, Fricke, Hetherington, Stone, & Chapman, 2010), which then persists for 12 hr (Rouse & Bretman, 2016). Males may be required to remember their recent social environment in order to determine whether the cues of competition have persisted for long enough to be representative of the general level of sperm competition (Rouse et al., 2018). The mechanisms by which long‐term memory facilitates responses to rivals by male *D. melanogaster* are localized to the mushroom bodies, highlighting the importance of olfactory cues (Rouse et al., 2018). Olfactory stimuli have also been found to be of particular importance for learning in the nematode *Caenorhabditis elegans*, in which learned associations with a number of aversive odors are formed at varying speeds (Choi et al., 2018). Therefore, although the multiple cues of *D. melanogaster* rival presence appear to be redundant in eliciting a longer mating response by males, there may be underlying differences related to how these cues are processed, the associative memories they form and the speed with which this happens. Indeed, the removal of auditory or olfactory cues of rivals has been found to extend the time taken for a male *D. melanogaster* to respond (Rouse & Bretman, 2016). Increasing understanding of the role of social cues in learning may shed further light on the mechanisms by which reproductive plasticity is achieved (Rouse et al., 2018).

### 4.2 Fitness effects of the extension of male *D. melanogaster* mating duration following detection of rivals

There was no evidence that the extension in mating duration following detection of competitors led to any immediate reproductive fitness benefits, either in the absence or presence of sperm competition. Neither males exposed to all rival cues, nor those for which auditory cues were removed, showed an increase in the number of offspring they fathered when the female was singly mated, compared to males experiencing no cues of competition. Males exposed to rivals, with all cues intact or with the auditory cue removed, also did not increase the proportion of paternity they achieved under sperm competition. In fact, in the absence of sperm competition, there was a trend for males not exposed to a rival to father more offspring than males previously exposed to rivals. In the first set of experiments, males in the − all treatment also achieved greater success in sperm competition than those in the + all treatment. Furthermore, the longer mating demonstrated by males exposed to rivals did not reduce female receptivity to remating.

The finding that exposure to rival males (either with all cues intact or with one cue removed) and the associated longer mating phenotype did not result in any apparent increases in reproductive fitness for *D. melanogaster* males was unexpected. Males exposed to cues of competition did not father higher numbers of offspring or reduce female receptivity to remating. This is inconsistent with previous findings (Bretman et al., 2010; Bretman, Westmancoat, et al., 2011). There is evidence that the fitness benefits associated with longer mating occur via increased transfer of two key seminal fluid proteins, sex peptide and ovulin, which increase female egg production and decrease receptivity to remating (Chapman et al., 2003; Chapman & Davies, 2004; Wigby, Sirot, et al., 2009). Neither of these effects were observed in the current study. Bretman et al. (2012) did find evidence that the behavioral response of longer mating duration can become decoupled from offspring production; however, this only occurred when males experienced a period without rival exposure prior to mating. Furthermore, Bretman et al. (2012) found evidence of males continuing to increase offspring production after mating duration was decreased, rather than of longer mating duration that did not correspond to fitness benefits. Nevertheless, Hopkins et al. (2019) found that sperm transfer and seminal fluid protein (SFP) transfer peak at different intensities of male–male competition, with the amount of SFPs transferred generally increasing with the level of competition. Additionally, the composition of SFPs in the ejaculate can change with the intensity of competition. These studies demonstrate that there may not be a simple relationship between level of competition, behavioral response, and reproductive success.

One possible explanation for the absence of an increase in reproductive success among males exposed to competitors is that aggressive interactions with rivals led to the treatment males sustaining harm, reducing their condition and thus their ability to increase their ejaculate investment in response to competition. Nandy, Dasgupta, Halder, and Verma (2016) found the expression of male–male aggression to be a key component of the cost of reproduction and a driver of decreased longevity under starvation in *D. melanogaster*. It has been proposed that aggression between males can impose costs via injury and energy expenditure (Bretman, Westmancoat, Gage, et al., 2013), ultimately reducing life span (Costa, Mateus, Moura, & Machado, 2010; Gaskin, Futerman, & Chapman, 2002). Males who suffer these costs from aggressive interactions during rival exposure may be less able to subsequently increase their investment in their ejaculate, negating the usual fitness benefits of extending mating duration. However, it has been argued that male–male aggression is a minor contributor to costs of reproduction (Bretman, Westmancoat, Gage, et al., 2013; Leech, Sait, & Bretman, 2017). This is based on the findings that males housed with a rival sustained no more wing damage than males housed alone (Bretman, Westmancoat, Gage, et al., 2013). Although social contact between male *D. melanogaster* does reduce lifespan, this could not be explained in any signature of behavioral differences between males (Leech et al., 2017). Moreover, male *Drosophila* aggression has been found to decline with prolonged exposure to the male‐specific pheromone 11‐*cis*‐vaccenyl acetate (cVA), suggesting that continuous exposure to rivals may reduce aggressive behavior. Thus, males housed with rivals may not be engaged in high frequencies of aggressive encounters, reducing the likelihood that they would sustain harm during treatment that would decrease their reproductive success.

Male competitive success can respond to various features of the social environment in addition to the presence of competitors, including female condition and female mating status (Bonduriansky, 2001; Friberg, 2006; Lewis & Iannini, 1995). The ejaculate investment of male *D. melanogaster* in this study may respond to these other variables, masking responses to the presence of competitors. This may explain why there was no elevation in offspring production from longer matings following exposure to rivals. For example, all females in this experiment were virgins prior to mating with the treatment males. Friberg (2006) found that males increased their investment in reproduction, leading to reduced female remating, when they perceived females to have previously mated. The virgin status of females in the current study may have cued to males the low probability of sperm competition, confounding the effects of the prior exposure to rivals. Furthermore, the virgin females in this study may have detected CHC components of previously encountered rivals on males in the + all and + no sound treatments, signaling the presence of other potential mates, while the females that mated with – all males may have inferred that this was the only likely mating opportunity. Therefore, females mating with the – all treatment males may have increased their per‐mating investment, thereby counterbalancing any increase in offspring production elicited by the males responding to rival cues. An alternative explanation for the uniformity in reproductive success across treatment groups is that all males were also virgins prior to the experimental mating. In polyandrous butterfly species, the first ejaculate a male produces is larger and contains more protein than subsequent ejaculates (Bissoondath & Wiklund, 1996; Hughes, Chang, Wagner, & Pierce, 2000). The male *D. melanogaster* tested here had not encountered a female since reaching reproductive maturity. Due to the high variation in the reproductive success of males (Bateman, 1948) and the very high potential fitness cost of never mating at all, it may be beneficial for a male to invest heavily in the first reproductive encounter, whether competition is detected or not. This too may have obscured the differences between treatment groups in reproductive success.

The apparent lack of fitness benefits of extending mating duration in response to rivals could occur because longer matings conferred benefits to males in the form of increased sperm displacement, which was not measured in this study. Reproductive success under sperm competition was only measured in terms of sperm defensiveness. However, previous research has found extended mating and increased reproductive success to follow exposure to rivals whether the focal male was the first or second to mate with a female. Another possible “hidden” fitness benefit of extending mating duration is the delaying of female remating up to 24 hr. Females were isolated for 24 hr following the first mating; thus, their receptivity to remating during this window was not measured. Reduced receptivity during the first 24 hr after mating could contribute to the adaptive value of increasing mating duration following rival exposure, despite the apparent lack of increase in offspring production. Nevertheless, it has been shown that behavioral responses to rivals can be decoupled from fitness benefits (Bretman et al., 2012; Hopkins et al., 2019). Furthermore, a recent study on *D. melanogaster* similarly found that longer matings by males exposed to competitors did not correspond to increased paternity share (Marie‐Orleach, Sanz, Bailey, & Ritchie, 2020).

Gilchrist and Partridge (2000) proposed that sperm transfer is completed within the first few minutes of *D. melanogaster* matings and therefore that longer matings do not correspond to increased sperm allocation, higher offspring numbers, or improved sperm displacement ability. However, interrupting matings past the point where sperm transfer would be completed impeded the male's ability to delay female remating, suggesting that some SFPs may continue to be transferred later in copulation. Furthermore, the sperm and SFP components of the ejaculate have been found to be under independent control. While sperm transfer peaks at a low level of sperm competition intensity, SFP transfer generally peaks under more intense competition, although the composition of proteins in the ejaculate may vary along the sperm competition gradient. (Hopkins et al., 2019). Mating duration therefore seems not to correspond to sperm transfer and may not be an appropriate proxy for overall ejaculate investment.

Together with our results that extended mating duration did not have any observed fitness benefits, these findings suggest that the relationship between cues of competition, behavior, and reproductive success may not be as simple or direct as previously thought. This opens further questions on how sensory cues are processed to infer the intensity, as well as risk, of sperm competition, and whether redundancy among cues persists at varying degrees of competition.

## 5 CONCLUSIONS

The results supported the previous finding that removing one cue of rival presence does not prevent male *D. melanogaster* from detecting rivals and responding to them by extending mating duration (Bretman, Westmancoat, et al., 2011). This suggests that the cues signaling rival presence are at least partially redundant. The redundancy of cue components may confer benefits to the receiving male by preventing information from being compromised if one component is inaccurate or lost, thereby facilitating adaptive reproductive plasticity. It cannot be concluded whether alternative combinations of cue components signaling rival presence are equivalent in terms of the fitness benefits achieved by responding to them, as no increase in reproductive success among males exposed to a rival was detected. Males exposed to all rival cues or the restricted set of cues did not increase their paternity, either in the absence or presence of sperm competition, despite extending mating duration. The receptivity of females to remating was also not affected by male exposure to rival cues. The absence of any apparent fitness benefits of longer mating is inconsistent with previous studies (Bretman et al., 2010; Bretman, Westmancoat, et al., 2011), but highlights that caution should be taken when indirectly extrapolating fitness benefits from behavioral responses alone. It is possible that the lack of increased offspring production following longer mating was caused by damage sustained from aggressive interactions with rivals impairing the male's ability to increase ejaculate investment. Or, the fitness benefits of longer mating may have been obscured by homogenizing effects other features of the social environment, such as male and female mating status. Alternatively, longer mating following rival exposure could have conferred “hidden” fitness benefits not measured in this study, for example sperm displacement or delaying of female remating up to 24 hr. However, it is also possible that behavior can become decoupled from increases in the transfer of sperm and SFPs and that this may be mediated the degree of male–male competition (Hopkins et al., 2019).

## CONFLICTS OF INTEREST

The authors declare no conflicts of interest.

## AUTHOR CONTRIBUTION

Alice A. Dore: Conceptualization (supporting); Data curation (equal); Formal analysis (lead); Investigation (lead); Methodology (equal); Validation (supporting); Visualization (lead); Writing‐original draft (equal); Writing‐review & editing (equal). Amanda Bretman: Conceptualization (equal); Formal analysis (supporting); Funding acquisition (supporting); Investigation (equal); Methodology (equal); Project administration (supporting); Supervision (supporting); Validation (supporting); Writing‐original draft (supporting); Writing‐review & editing (supporting). Tracey Chapman: Conceptualization (lead); Data curation (equal); Formal analysis (equal); Funding acquisition (lead); Investigation (equal); Methodology (equal); Project administration (lead); Resources (lead); Supervision (lead); Validation (equal); Visualization (equal); Writing‐original draft (equal); Writing‐review & editing (equal). 

## Supporting information

Figure S1

Figure S2

Figure S3

Figure S4

Supplementary Material

## Data Availability

All the raw data associated with this publication are deposited in the DRYAD database, https://doi.org/10.5061/dryad.v9s4mw6rt
