# Supplementary figures and images for: Fitness consequences of redundant cues of competition in male Drosophila melanogaster
Source: Ecol Evol. 2020 May 4;10(12):5517–26. doi: 10.1002/ece3.6293 (PMC7319233; doi:10.1002/ece3.6293)

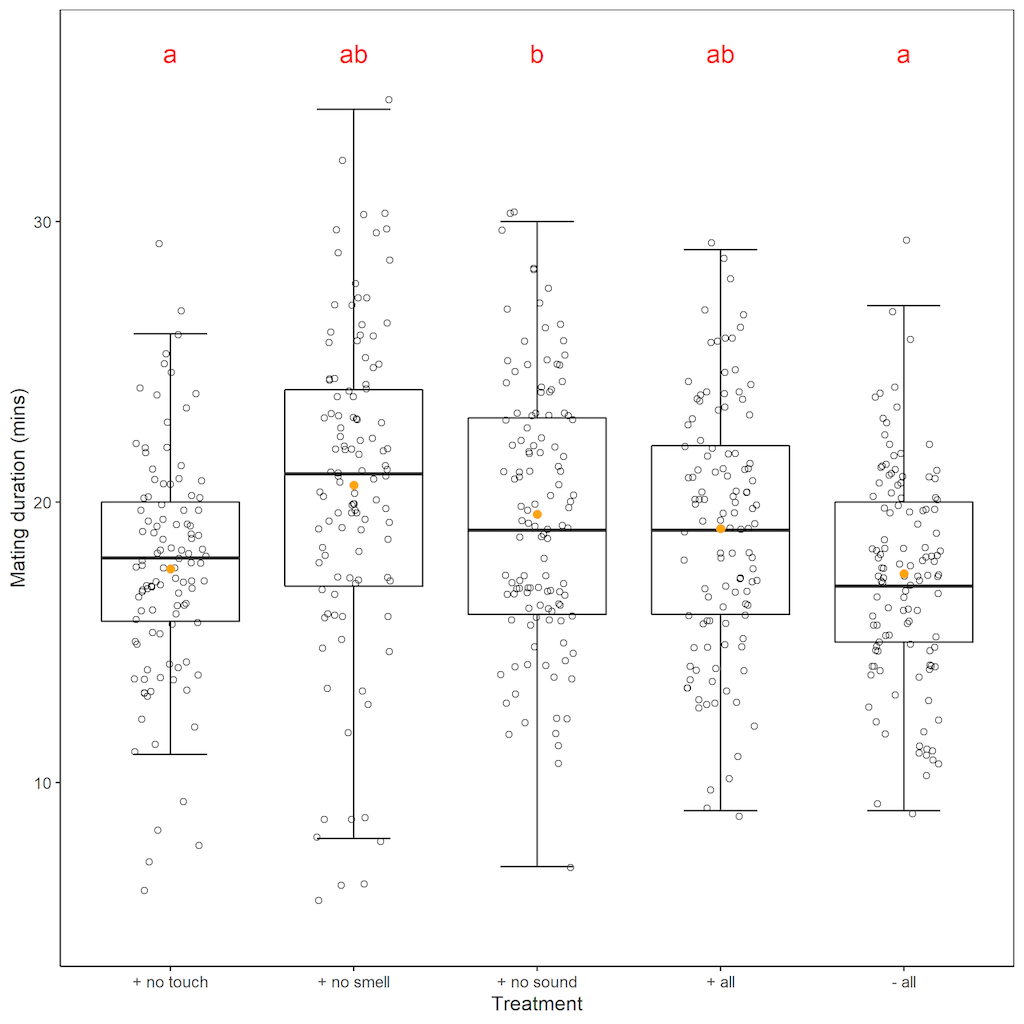

Supplement: Supplementary file 1 — Figure S1 [file ECE3-10-5517-s001.tiff]

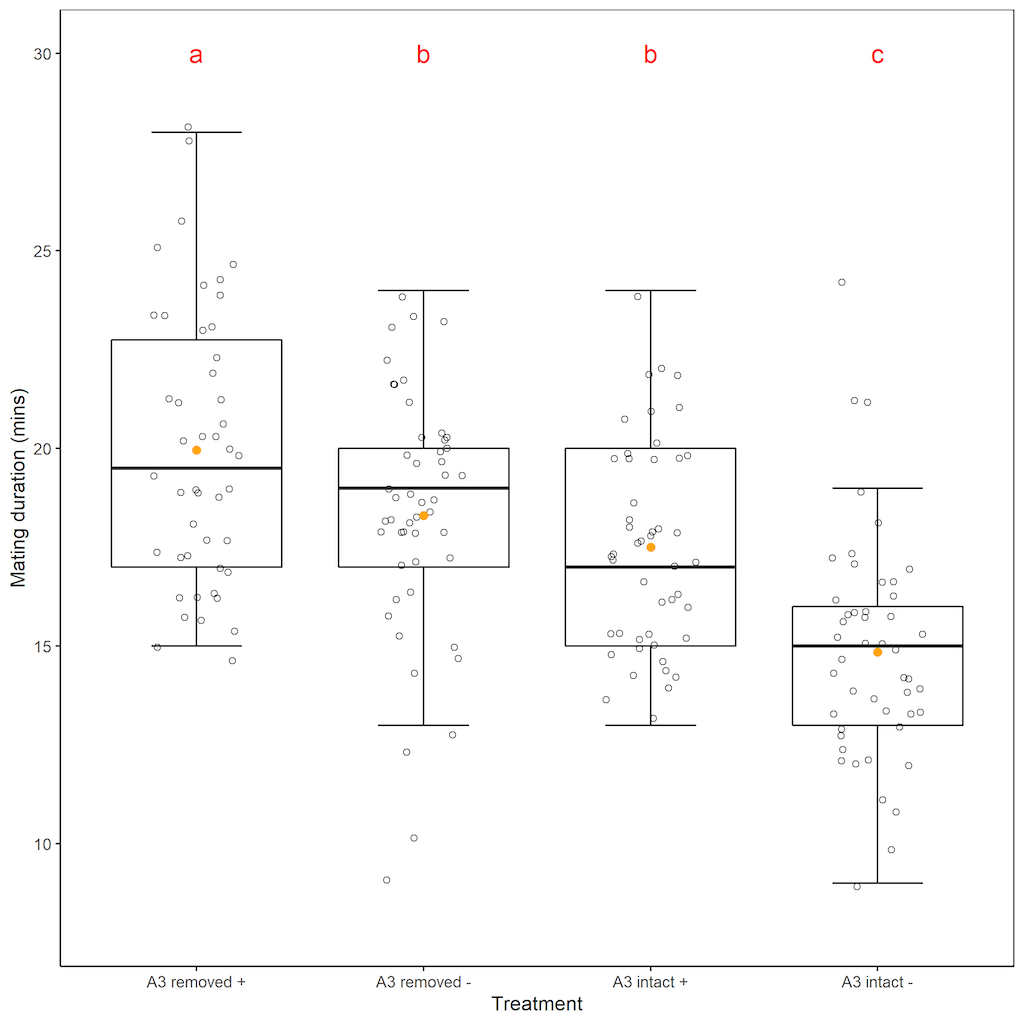

Supplement: Supplementary file 2 — Figure S2 [file ECE3-10-5517-s002.tiff]

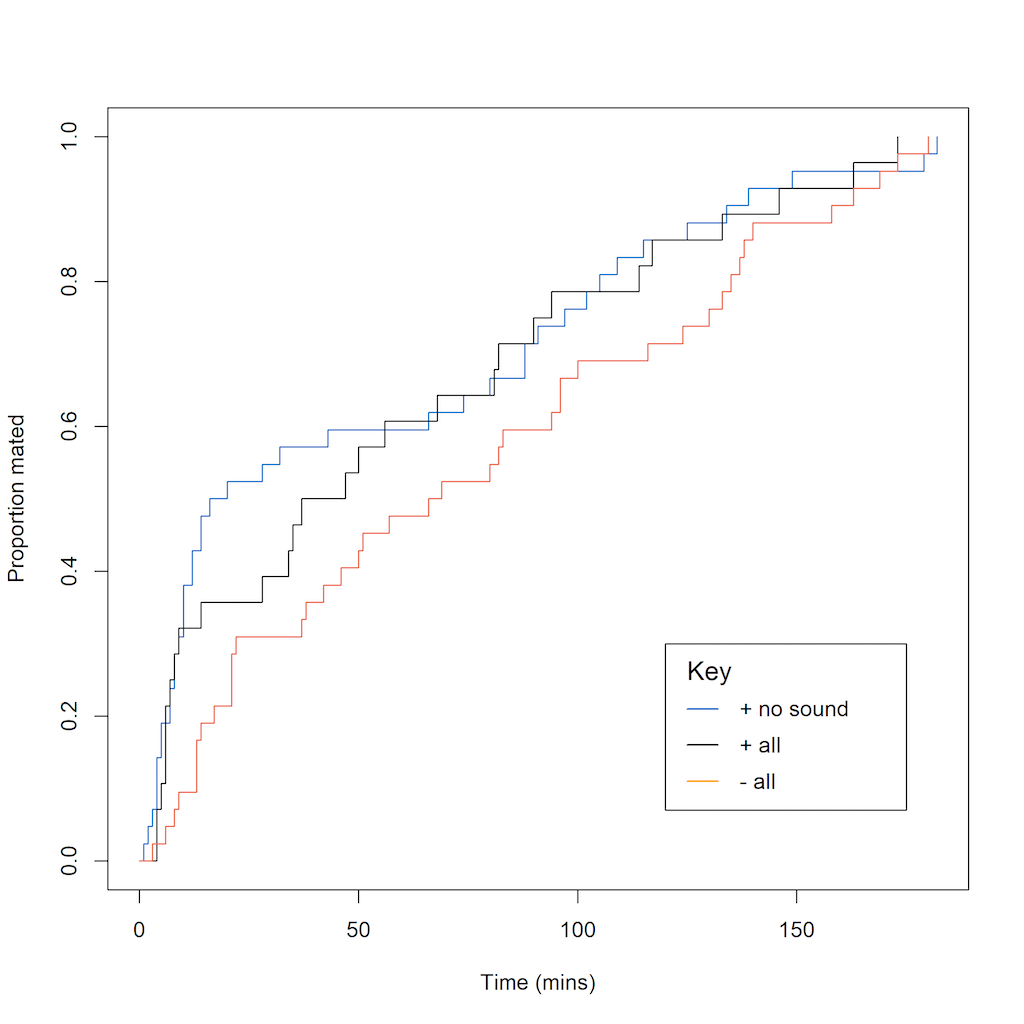

Supplement: Supplementary file 3 — Figure S3 [file ECE3-10-5517-s003.tiff]

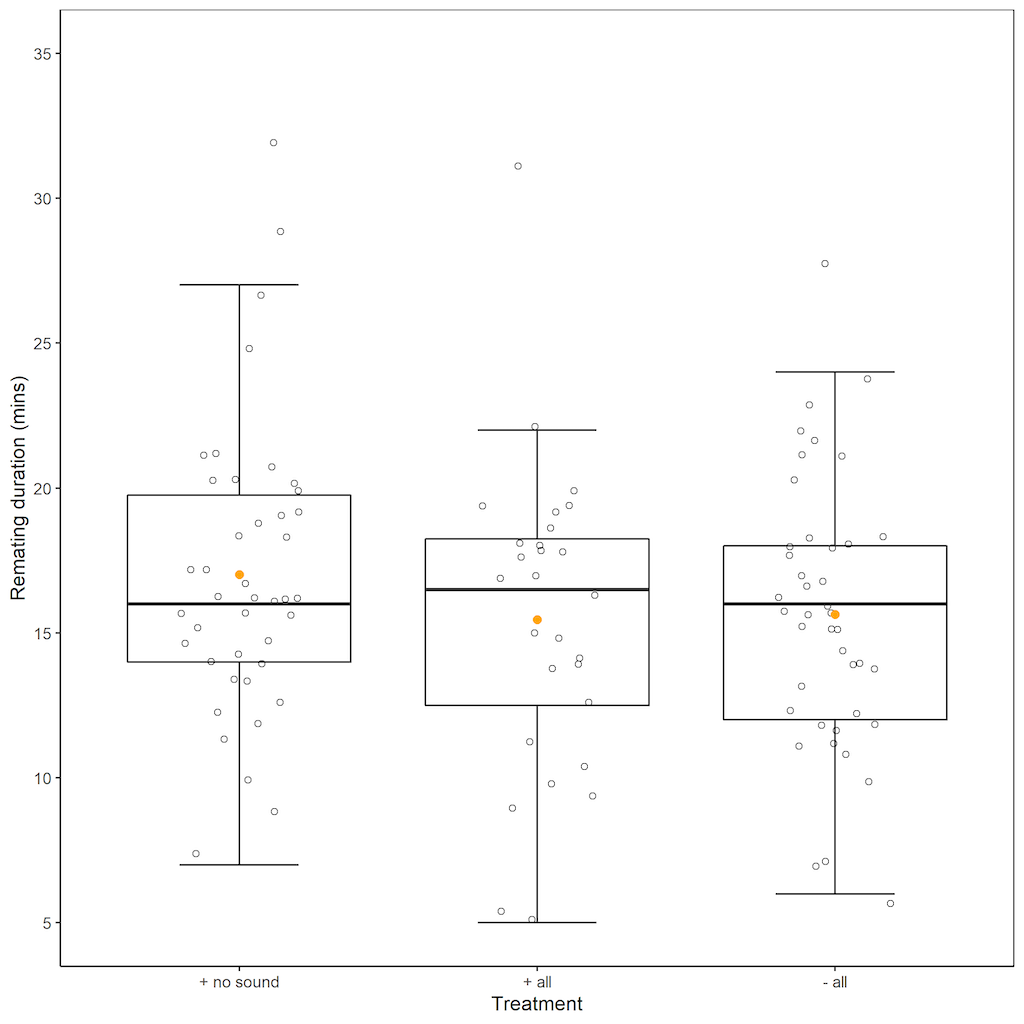

Supplement: Supplementary file 4 — Figure S4 [file ECE3-10-5517-s004.tiff]
