# Supplementary material for: Fitness consequences of redundant cues of competition in male Drosophila melanogaster
Source: Ecol Evol. 2020 May 4;10(12):5517–26. doi: 10.1002/ece3.6293 (PMC7319233; doi:10.1002/ece3.6293)
Supplement: Supplementary file 5 — Supplementary Material [file ECE3-10-5517-s005.docx]

Appendix

1. Effect of systematic removal of auditory, olfactory and tactile cues of *D. melanogaster* rival presence

Methods

1. General methods

As described in main text.

1. Removal of cues of rival presence

Each male was randomly assigned to one of the following treatments: 1) housed with a wildtype rival male with the tactile cue removed (+ no touch), 2) housed with a rival male with the olfactory cue removed (+no smell), 3) housed with a rival male with the auditory cue removed (+no sound), 4) housed with a rival male with all sensory cues intact (+all), 5) housed alone (-all). The auditory cue of rival presence was inhibited via the removal of the wings of rival males, as described in the main text. To control for handling and allow identification of the focal male, the rival males in the +no touch, +no smell and +all treatments were also subjected to CO_2_ anaesthesia and their wings were clipped. The focal and rival males in the + no sound and + all treatments were housed together in a single SYA vial. To remove the tactile cue in the + no touch treatments, the focal and rival male were placed in separate vials of SYA which were joined together at their open ends, with porous nylon netting between the two. Through the netting, the two males were expected to be able to smell and hear each other, but not touch. The males in the - all treatment were housed alone in a vial. To inhibit the olfactory cue of rival presence, the third antennal segments (A3) of the focal males in the +no smell treatment were removed under CO_2_ anaesthesia. Focal males in all other treatments were also briefly anaesthetised with CO_2_ to control for experience. Focal males were maintained in their respective treatments for three days. Two independent replicate experiments were carried out and the data were pooled for analysis with replicate as a fixed factor.

1. Effect of cue removal on responses to rivals

A mating assay was carried out as described in the main text to measure latency to mate and mating duration.

1. Statistical analyses

As described in the main text, mating duration and latency were modelled in R v 3.4.2 (R Core Team 2016) using mixed models. Mating duration was modelled with a linear model. Mating latency data were analysed using a cox proportional hazards model. Post-hoc pairwise comparisons were conducted using the package ‘multcomp’ (Hothorn et al. 2008).

Results & discussion

The combination of sensory cues of rival presence to which the focal male was exposed significantly affected mating duration (F=9.62, df=4 & 566, p<0.001; Figure S1). Latency to mate varied significantly between replicates (X^2^=22.72, df=1, p<0.001), but was not significantly affected by the rival cues the focal male encountered (X^2^=2.29, df=4, p=0.68).

When all cues were intact (+ all) males did not mate for significantly longer than males with no rival exposure (- all; p=0.059). The significant extension of mating duration by male *D. melanogaster* in response to rivals has previously been found to be a robust and repeatable result (Bretman et al. 2009; Bretman et al. 2011a; Bretman et al. 2011b). Therefore, the nonsignificant difference in mating duration between + all and – all treatments in this study suggested confounding factors or imprecision in the data, which complicated the interpretation of the results. Nevertheless, males with no rival exposure expressed significantly shorter mating than males exposed to rivals with either the olfactory (p<0.001) or the auditory (p=0.0036) cue removed. Males exposed to rivals with the tactile cue removed also mated for significantly shorter duration than those with the olfactory (p<0.001) or auditory (p=0.012) cue occluded. This suggested that the removal of the tactile cue prevented males from responding to rivals by extending mating duration, instead showing similar mating duration to males that had not encountered rivals. To prevent focal and rival males from touching, the two were housed in separate vials with porous netting between them. This treatment differed from the other protocols to remove a cue, in which males were housed together in a single vial. This may have had resulted in confounding influences on the perception of the rival, for example by diluting the olfactory and auditory cues. Therefore, this treatment was not included in subsequent experiments, to avoid off-target effects.

2. Effect of inhibiting olfactory cue of *D. melanogaster* rival presence by removal of the A3 antennal segment

Methods

1. General methods

As described in main text.

1. Effect of antennal segment removal on responses to rivals

Each focal male was randomly assigned to one of the following treatments: 1) A3 removed with subsequent rival exposure (A3 removed +), 2) A3 removed with no rival exposure (A3 removed -), 3) A3 intact with subsequent rival exposure (A3 intact +), 4) A3 intact with no rival exposure (A3 intact -). Focal males in the two A3 removed treatments were anaesthetised with CO_2_ and the third antennal segment was removed with sharpened forceps. To control for handling, focal males in the A3 intact treatments were also anaesthetised with CO_2_. To distinguish rival males from focal males, the wings of rival males were clipped under CO_2_ anaesthesia. Only the tips of the wings were removed, so as not to prevent the rival males from producing the song which functions as the auditory cue of their presence (Bretman et al. 2011b). Each focal male in the A3 removed + and A3 intact + treatments was housed with a conspecific wildtype rival male in a vial of SYA for three days. Males in the A3 removed – and A3 intact – treatments were housed alone in a vial of SYA during this time.

A mating assay was carried out as described in the main text to measure latency to mate and mating duration.

1. Statistical analyses

Statistical analyses were carried out in R v 3.4.2 (R Core Team 2016). Mating duration data were analysed using a linear model. Mating latency data were analysed using a cox proportional hazards model. Post-hoc Tukey tests pairwise tests were carried out using the ‘multcomp’ package (Hothorn et al. 2008).

Results & discussion

The removal of A3 significantly influenced both mating duration (F=44.74, df=1 & 190, p<0.001; Figure S2) and latency to mate (X^2^=9.92, df=1, p=0.0016). No significant response to rivals in mating latency was identified within either the A3 removed or the A3 intact treatments. Despite the overall significant effect of A3 removal on mating latency, there were no significant pairwise differences between males with A3 removed or A3 intact in equivalent rival treatments. Both males with A3 removed (p=0.045) and males with A3 intact (p<0.001) significantly extended mating duration following exposure to rivals, compared to males housed alone. Males in the A3 removed + treatment mated for significantly longer than the A3 intact + treatment (p<0.001). Males in the A3 removed - treatment also significantly extended mating duration, relative to the A3 intact - treatment (p<0.001). This showed that surgical A3 removal did affect the behaviour of male *D. melanogaster* - therefore this manipulation did not cleanly remove the olfactory cue of rival presence without extraneous effects.
